# Construction of a lipid metabolism-related and immune-associated prognostic score for gastric cancer

**DOI:** 10.1186/s12920-023-01515-w

**Published:** 2023-05-03

**Authors:** Jing Dai, Qiqing Li, Jun Quan, Gunther Webb, Juan Liu, Kai Gao

**Affiliations:** 1 Department of Gastrointestinal Surgery, The Third Xiangya Hospital of Central South University, Changsha, 410013 Hunan People’s Republic of China; 2 Department of Dermatology, The Second Xiangya Hospital, Central South University, Changsha, 410011 Hunan People’s Republic of China

**Keywords:** Lipid metabolism, Prognosis, Gastric cancer, Immune infiltration, ST6GALNAC3

## Abstract

**Background:**

The interaction between tumor cells and immune or non-immune stromal cells creates a unique tumor microenvironment, which plays an important role in the growth, invasion and metastasis of gastric cancer (GC).

**Methods:**

The candidate genes were selected to construct risk-score by univariate and multivariate Cox regression analysis. Nomograms were constructed by combining clinical pathological factors, and the model performance was evaluated by receiver operating characteristic curve, decision curve analysis, net reclassification improvement and integrated discrimination improvement. The functional enrichment between high-risk group (HRisk) and low-risk group (LRisk) was explored through GO, KEGG, GSVA and ssGSEA. CIBERSORT, quanTIseq and xCell were used to explore the immune cell infiltration between HRisk and LRisk. The relevant EMT scores, macrophage infiltration scores and various metabolic scores were calculated through the “IOBR” package and analyzed visually.

**Results:**

Through univariate and multivariate Cox regression analysis, we obtained the risk-score of fittings six lipid metabolism related genes (LMAGs). Through survival analysis, we found that risk-score has significant prognostic significance and can accurately reflect the metabolic level of patients. The AUCs of the nomogram model incorporating risk-score 1, 3 and 5 years were 0.725, 0.729 and 0.749 respectively. In addition, it was found that the inclusion of risk-score could significantly improve the prediction performance of the model. It was found that the arachidonic acid metabolism and prostaglandin synthesis were up-regulated in HRisk, and more tumor metastasis related markers and immune related pathways were also enriched. Further study found that HRisk had higher immune score and M2 macrophage infiltration. More importantly, the immune checkpoints of tumor associated macrophages involved in tumor antigen recognition disorders increased significantly. We also found that ST6GALNAC3 can promote arachidonic acid metabolism and up-regulate prostaglandin synthesis, increase M2 macrophage infiltration, induce epithelial mesenchymal transformation, and affect the prognosis of patients.

**Conclusions:**

Our research found a novel and powerful LMAGs signature. Six-LMAGs features can effectively evaluate the prognosis of GC patients and reflect the metabolic and immune status. ST6GALNAC3 may be a potential prognostic marker to improve the survival rate and prognostic accuracy of GC patients, and may even be a potential biomarker of GC patients, indicating the response to immunotherapy.

**Supplementary Information:**

The online version contains supplementary material available at 10.1186/s12920-023-01515-w.

## Introduction

Gastric cancer (GC) is the fifth most common malignant tumor and the third leading cause of cancer-related death in the world [1]. East Asia, including China, has the highest mortality rate [2, 3]. Although the overall survival rate has improved with the progress of surgical treatment, chemotherapy and targeted therapy, the survival rate of GC patients is still less than 30% [4, 5]. At present, the specific mechanism of GC development is still unclear. Recent evidence shows that the interaction between tumor cells and immune or non-immune stromal cells creates a unique tumor microenvironment, which plays a crucial role in tumor growth, invasion and metastasis [6, 7].

Epithelial mesenchymal transformation (EMT) of cancer cells has been identified to play a key role in tumor progression, invasion and metastasis, and is a way for cancer cells to gain more aggressiveness. In the process of EMT, epithelial cells decreased the expression of epithelial markers (E-cadherin) and took place phenotypic transformation, and increased the expression of mesenchymal markers (N-cadherin, VIM, ZEB1), thus showing decreased intercellular adhesion and increased motility [8, 9]. Enhanced motility and invasiveness provided by EMT are crucial for the metastasis of cancer progression, and the acquisition of mesenchymal phenotype has been proved to enhance the resistance to chemotherapy and lead to poor prognosis [10, 11]. Studies have shown that lipid metabolism reprogramming plays a key role in the process of EMT.

Lipids not only play a key role in maintaining cell membrane homeostasis, but also in signal transduction. More and more evidence show that lipid metabolism is an important regulator of cancer progression and EMT. Eicosanoid, including prostaglandins (PGs), leukotrienes (LTS) and lipoxins (LXS), are signal molecules produced mainly through the oxidation of arachidonic acid (AA) [12–14]. AA derived eicosanoid plays a complex role in controlling a wide range of physiological processes, including cytokine production, antibody formation, differentiation, cell proliferation, migration and antigen presentation [15]. Prostacyclin can promote cancer development through a variety of mechanisms, including regulating tumor epithelial cell biology and promoting tumor related angiogenesis. PGE2 catalyzed by COX2 plays a key role in the invasion of ovarian cancer cells [16], and can promote the proliferation of cancer cells in vitro and in vivo [17, 18]. PGE2 promotes tumor invasion and metastasis by activating PI3K/Akt/mTOR pathway and JAK2/STAT3 pathway [19]. COX-2-derived PGE2 can stimulate cell proliferation, angiogenesis and enhance cell invasiveness [20], but the mechanism of inhibiting tumor immunity is not clear.

Tumor-associated macrophage (TAMs) play an important role in tumor micro-environment (TME) of various solid malignancies. The number of infiltrated M2 macrophages and total TAM may be factors of poor prognosis in patients with gastric cancer, while M1 macrophage infiltration is associated with better survival [21]. At the same time, the phenotypic changes of macrophages are closely related to the lipid metabolism reprogramming of cancer cells, and guide macrophages to play a completely different immune function. Studies have shown that eicosanoid can regulate the inflammatory function of macrophages. For example, macrophage derived PGs limit TNF-a production in an autocrine manner. The effect of PGE2 on macrophages is to inhibit Th1 immune response [22]. However, the mechanism of eicosanoid on macrophage polarization is not clear.

Activated immune cells and cancer cells have similar metabolic pathways in some aspects, but they also compete for basic nutrients in TME. In addition, it is found that the metabolic pathway of immune cells is closely related to their immune function. Therefore, understanding the major metabolic differences between gastric cancer cells and immune cells activated in TME to connect intratumoral metabolism and immunotherapy may be a potential mechanism to enhance tumor immunotherapy. Therefore, we established a novel lipid metabolism score and constructed a nomogram in combination with clinicopathological factors to predict patient overall survival. We further explored the specific mechanism of lipid metabolism reprogramming promoting the progression of gastric cancer, and explored the relationship between lipid metabolism reprogramming and immune cell infiltration; Finally, we identified ST6 N-acetylgalactosaminide alpha-2,6-sialyltransferase 3 (ST6GALNAC3) as a potential therapeutic target related to lipid metabolism, which will contribute to the immunotherapy of gastric cancer.

## Methods

### Patient cohort and data preparation

The selection criteria for this study are as follows: (1) definite histological diagnosis of GC; (2) definitive clinical data; (3) at least 30 days of overall survival after initial pathologic diagnosis [23]; (4) complete RNA-seq data. RNA-seq data and related clinical data were downloaded from TCGA (Additional file 1: Fig. S1) and GEO databases. TCGA data included 335 GC samples. GSE84437 contains 426 GC samples (Additional file 2: Table S1). In addition, we collected 243 lipid metabolism-associated genes (LMAGs) based on the Molecular Signatures Database v7.0.8,9 [24]. 19 LMAGs were identified as prognostic by using univariable Cox regression analysis. Candidate genes for constructing the risk model were selected by multivariate Cox stepwise regression analysis.

### Evaluation of risk model independence

The patients were divided into low-risk (LRisk) and high-risk group (HRisk) with the median risk score as the cutoff. Kaplan–Meier analysis was used to estimate the difference in overall survival among classified patients. Then, to combine the OS-model with clinicopathological data, the nomograms were developed by “regplot” package. In addition, the area under the curve (AUC) of the receiver operating characteristic (ROC) was calculated to evaluate the predictive ability of nomogram or other models. We also utilized the decision curve analysis (DCA) to evaluate the potential clinical effects of models. We calculated the expression of LMAGs in single cells using TISCH database, and explored the prognostic significance of LMAGs using OncoLnc and HPA database.

### Functional analysis

Based on GSVA score, "LIMMA" R software package was used to analyze the difference REACTOME path between the two groups of HRisk patients in the two cohorts. Gene ontology (GO) and KEGG analyses were performed to enrich the DEGs into associated pathways using the “clusterProfiler” R package [25]. In addition, in order to estimate the activation degree of 50 HALLMARK pathways, "ssGSEA" R package were applied under the standard setting.

### Tumor immune microenvironment analysis

The immune score, stromal score and tumor purity of patients were calculated by R package "estimate". Ciberport, quantiseq and xcell algorithms were used to estimate the degree of immune cell infiltration. The correlation between LMAGs and immune cells was calculated by TIMER and TIMER2.0. We calculated relevant EMT scores according to the work of Powles et al. [26], macrophage infiltration scores according to the work of Rooney [27], Danaher [28], Bindea [29] and Peng [30] et al., and various metabolic scores according to the work of smiraglia et al. [31], the above algorithms are included in the “IOBR” R package, and finally calculated and visualized with “IOBR” R package [32].

### RNA isolation and quantitative real-time RT-PCR

We performed experiments with reference to previous studies [33]. AGS GC cells were obtained from Zhongqiao New Prefecture in Shanghai. All cells were grown in medium containing 10% fetal bovine serum (FBS; Gibco, NY, USA) and 1% penicillin–streptomycin (HyClone, Logan, UT, USA) in a standard humidified incubator. Total RNA was extracted using TRIzol Reagent (TaKaRa, Beijing, China) and reverse transcribed into cDNA using the PrimeScript RTMaster Mix (Perfect Real Time) reagent (TaKaRa, Beijing, China) according to the manufacturer’s instructions. Quantitative real-time PCR (qRT-PCR) was performed on an ABI 7500HT Fast Real-Time PCR System (Applied Biosystems, CA, USA). The average fold of relative mRNA expression was determined using the 2^−ΔΔCt^ method with GAPDH as an internal control [33]. Primer sequences for qRT-PCR were as follows:

- ST6GALNAC3, forward 5′-ACCAGCGTTCCTCTTTTGCT-3′ and reverse 5′-TCATGCGCTTCTCTGTGGTC-3′;
- ROR2, forward 5′-AAGGAACCTCCCCAGCCA-3′ and reverse 5′-GCCACCACCCCTTTCTACG-3′;
- TAGLN, forward 5′-CCATGCCAGACAGCAGAGG-3′ and reverse 5′-ACTCTGCTTTGGAGTACAGCC-3′;
- GAPDH, forward 5′-TCGACAGTCAGCCGCATCTT-3′ and reverse 5′-GAGTTAAAAGCAGCCCTGGTG-3′.

### Statistical analysis

All statistical analyses were conducted by R software (“ggcorrplot” package and “ggstatsplot” package) (version 4.1.2). Survival curves were compared using log-rank test and performed using the Kaplan–Meier method. *P* < 0.05 (two-tailed) was considered statistically significant.

## Results

### Screening candidate genes for constructing risk-score

243 genes related to lipid metabolism were collected through KEGG database. We found that 19 genes related to lipid metabolism were meaningful through univariate Cox regression analysis (Additional file 3: Table S2). Then we performed multivariate Cox stepwise regression analysis on the 19 genes. Finally, we obtained the risk-score of fitting 6 genes related to lipid metabolism (ADH4, AKR1B1, CYP4A11, NEU2, SMPD3, ST6GALNAC3).

### Establishment and verification of risk-score

**Fig. 1:**
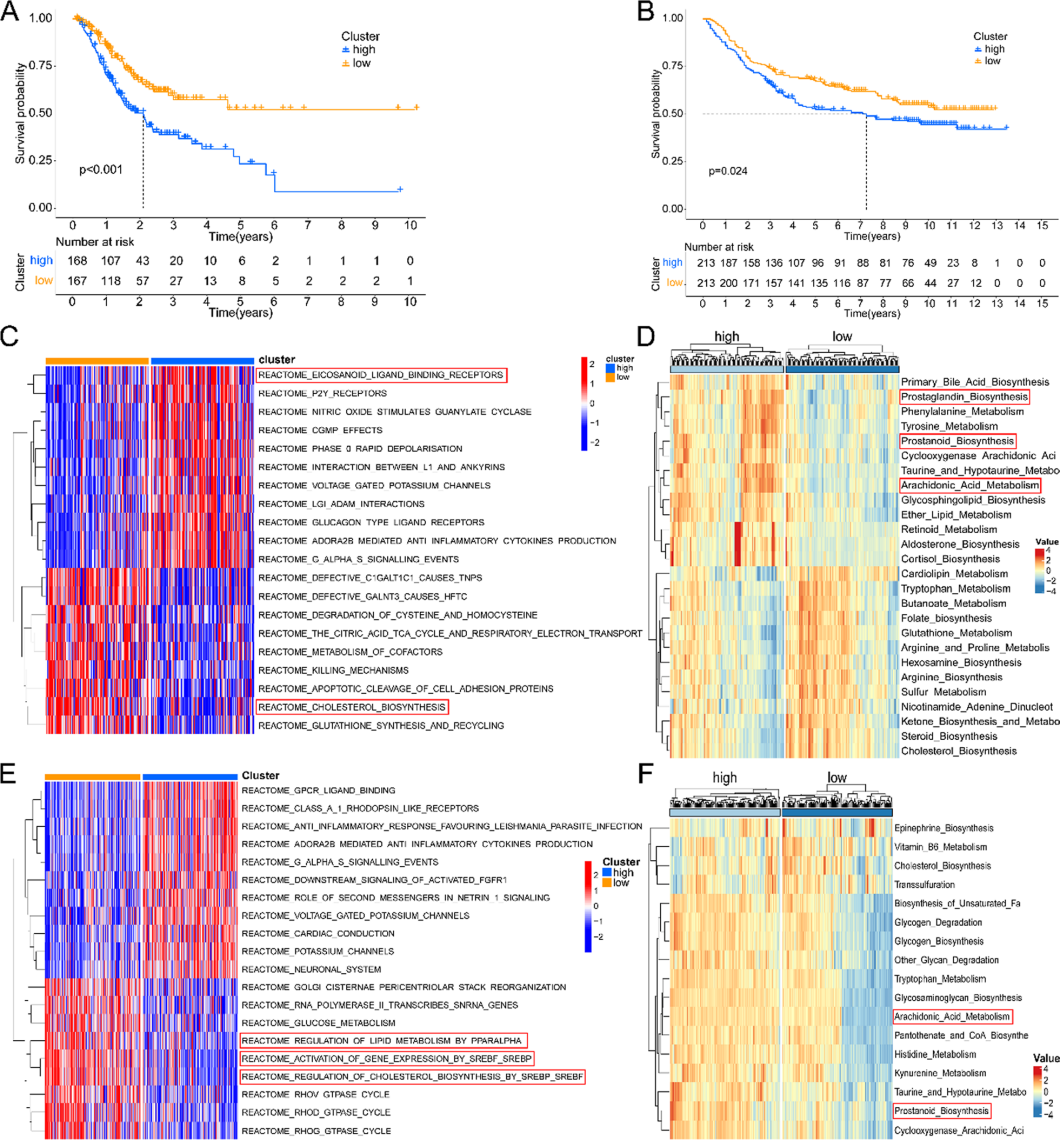
Establishment and verification of risk-score. Kaplan–Meier curve of HRisk and LRisk in TCGA (**A**) and GSE84437 (**B**). The enrichment of REACTOME database pathway between HRisk and LRisk was analyzed by GSVA in TCGA (**C**) and GSE84437 (**E**). The “IOBR” package calculates the subroutine reprogramming between HRisk and LRisk in TCGA (**D**) and GSE84437 (**F**)

Through survival analysis, we found that the risk-score has significant prognostic significance. The higher the risk-score, the lower the overall survival rate of patients (Fig. 1A, B). The metabolic programming between HRisk and LRisk was calculated by “IOBR” package. We found that there were significant differences in the expression of lipid metabolism related products between HRisk and LRisk (Fig. 1D and Additional file 1: Fig. S2A, Fig. 1F and Additional file 1: Fig. S2B). GSVA was used to calculate the difference between HRisk and LRisk in the REACTOME database (Fig. 1C, E). We found that there was a significant difference in the enrichment of lipid metabolism related pathways between HRisk and LRisk. We also verified it with GEO data and got the same conclusion. More importantly, we found that the HRisk group promoted arachidonic acid metabolism and prostanoid biosynthesis. Therefore, the lipid metabolism related score we constructed can accurately reflect the level of metabolism in patients.

### Establishment, evaluation and verification of Nomogram

**Fig. 2:**
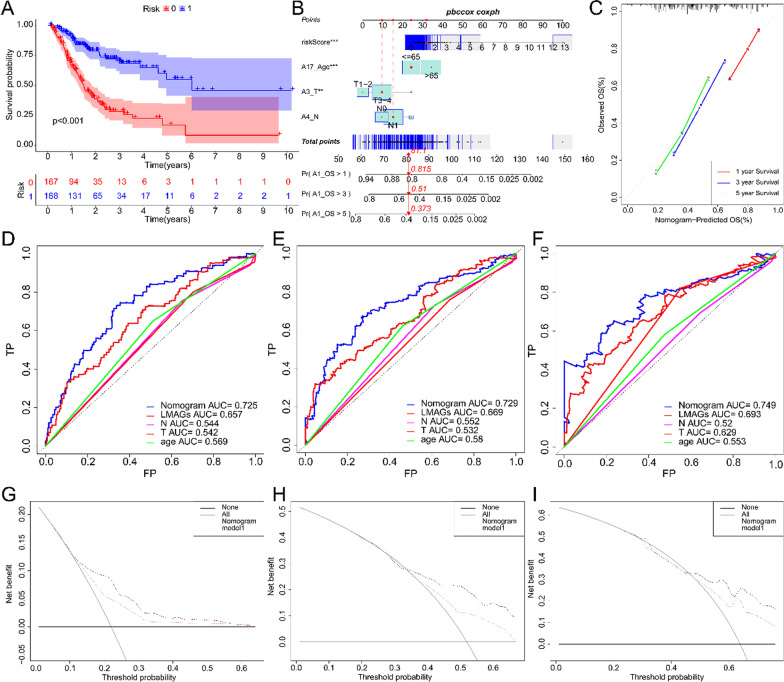
Establishment and evaluation of Nomogram. Kaplan–Meier curve of Nomogram in TCGA (**A**). Nomogram built according to TCGA (**B**). Nomogram calibration curve for 1, 3 and 5 years (**C**). Nomogram ROC curve for 1, 3 and 5 years (**D**–**F**). Nomogram DCA curve for 1, 3 and 5 years (**G**–**I**)

We combined risk-score and clinicopathological factors to conduct univariate Cox regression analysis, and found that the patient's age, T stage, N stage and risk-score were meaningful. Then we incorporated the meaningful indicators of univariate Cox regression analysis into the multivariate Cox regression, and built a model to predict the overall survival rate of patients (Fig. 2B and Additional file 1: Fig. S3A). We found that this model can accurately predict the prognosis of patients in training set and validation set (Fig. 2A). We visualized the model through nomogram, and found that nomogram have good consistency through calibration curve detection (Fig. 2C). According to ROC curve, the AUC of nomogram in 1, 3 and 5 years are 0.725, 0.729 and 0.749 respectively, indicating that nomogram have good prediction performance (Fig. 2D–F). In addition, we also found that risk-score has better predictive ability than conventional clinicopathological factors. The same is true in the validation set (Additional file 1: Fig. S3B-D). In addition, when the NRI and IDI were analyzed, which are more sensitive than the other methods used in this study, we found that including the risk-score can significantly improve the predictive accuracy of the nomogram models (Additional file 4: Table S3). We also used the DCA to assess the potential clinical effects of the nomograms with or without the risk-score (Fig. 2G–I). We found that nomogram with risk-score have better clinical decision-making ability. To sum up, we found that risk-score has a good ability to predict the overall survival of patients, and nomogram incorporated into risk-score have a good prediction performance.

### Potential functional analyses of the risk-score

**Fig. 3:**
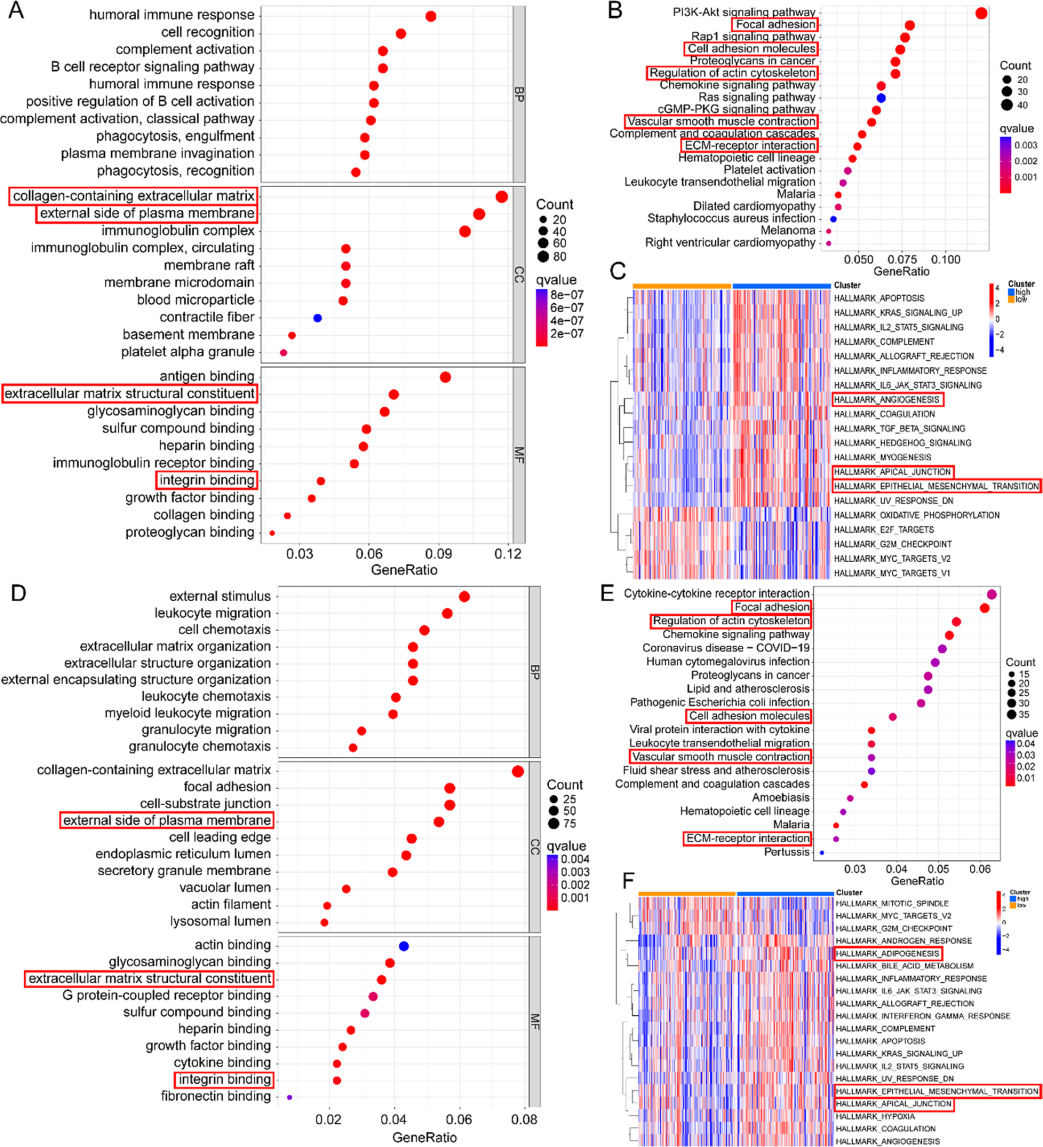
Potential functional analyses in TCGA and GSE84437. Go was used to analyze the enrichment between HRisk and LRisk (**A**). KEGG was used to analyze the enrichment between HRisk and LRisk (**B**). Analysis of Hallmark enrichment between HRisk and LRisk using ssGSEA (**C**). Go was used to analyze the enrichment between HRisk and LRisk (**D**). KEGG was used to analyze the enrichment between HRisk and LRisk (**E**). Analysis of Hallmark enrichment between HRisk and LRisk using ssGSEA (**F**)

**Fig. 4:**
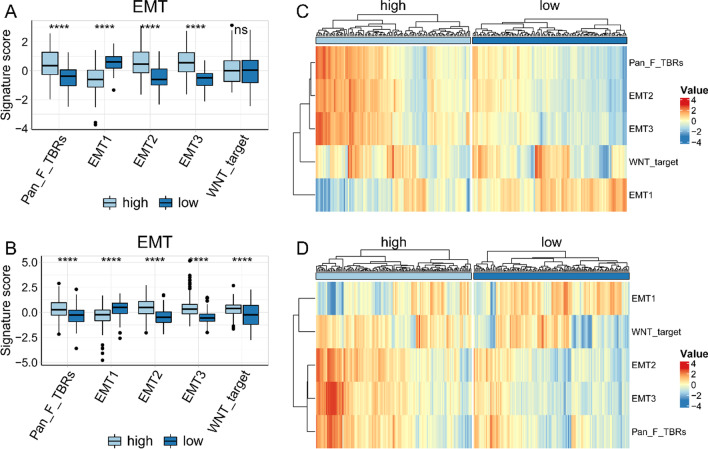
Relationship between risk-score and EMT score. The box chart shows the relationship between risk-score and EMT score in TCGA (**A**) and GSE84437 (**B**). The heat map shows the relationship between risk-score and EMT score in TCGA (**C**) and GSE84437 (**D**)

In order to further explore the biological function of lipid metabolism reprogramming, through GO analysis of the differential genes between HRisk and LRisk (Additional file 5: Table S4), we found that more immune related pathways were enriched in GOBP, and more extracellular matrix (ECM) related pathways were enriched in GOCC and GOMF (Fig. 3A, D). We also found that ECM, focal adhesion and vascular smooth muscle contraction were significantly enriched in KEGG database (Fig. 3B, E). More importantly, we analyzed the difference of Hallmark between HRisk and LRisk through ssGSEA, and found that angiogenesis and epithelial mesenchymal transition were significantly enriched in HRisk (Fig. 3C, F). We further analyzed the EMT related markers of Powles et al. And found that the patients in the HRisk group had higher EMT scores than those in the LRisk group, and were more prone to epithelial mesenchymal transition (Fig. 4A–D). In conclusion, the reprogramming of lipid metabolism may affect the invasion, metastasis and immune microenvironment of tumor cells.

### Correlation between risk-score and tumor immune microenvironment

**Fig. 5:**
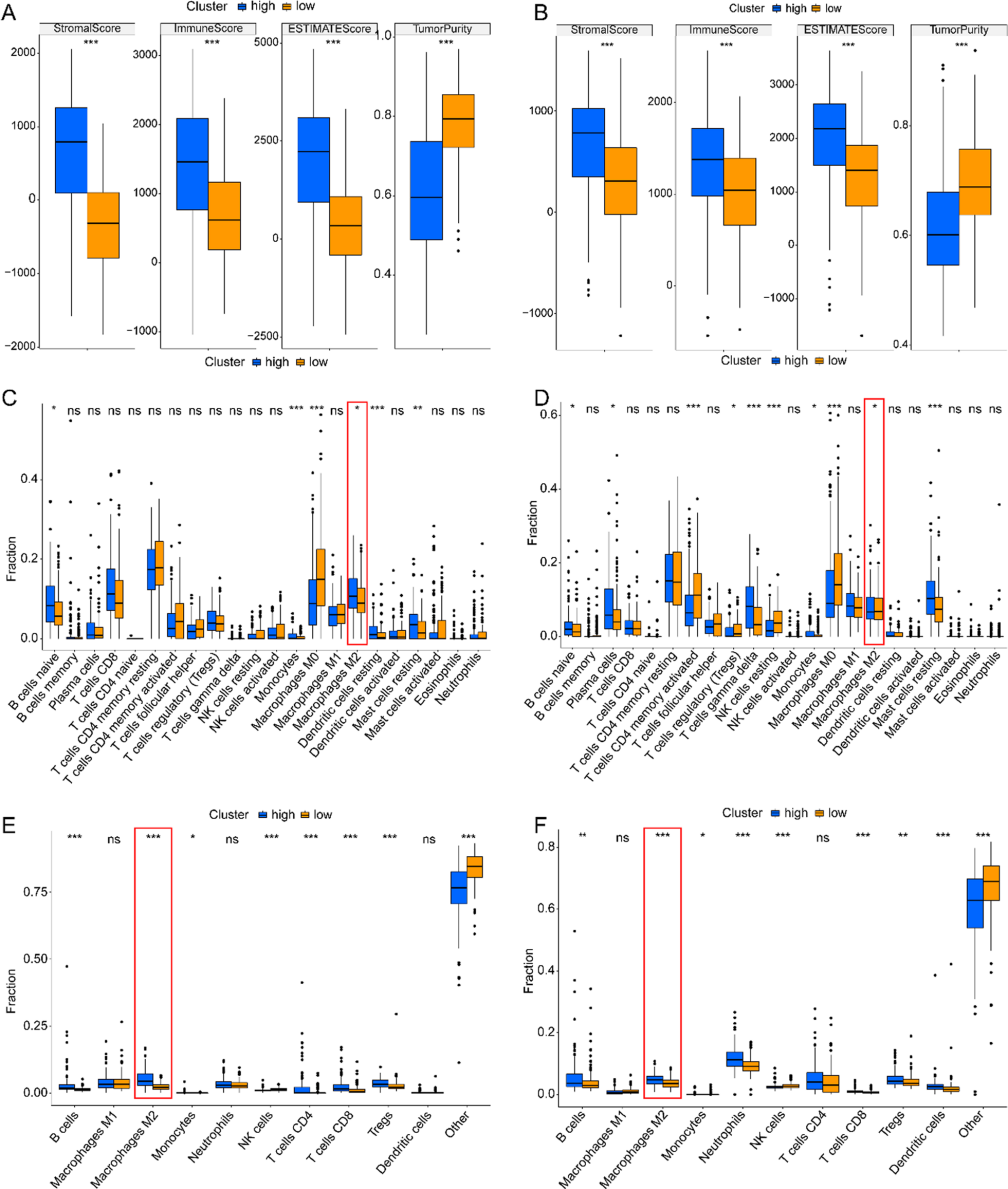
The relationship between risk-score and tumor immune microenvironment. Comparison of TumorPurity, ImmuneScore and StromalScore between the HRisk and LRisk patients in TCGA (**A**) and GSE84437 (**B**). Boxplots depicting the CIBERSORT scores of 22 immune cells of the HRisk patients compared to LRisk patients in TCGA (**C**) and GSE84437 (**D**). Boxplots depicting the quanTIseq scores of 11 immune cells of the HRisk patients compared to LRisk patients in TCGA (**E**) and GSE84437 (**F**)

**Fig. 6:**
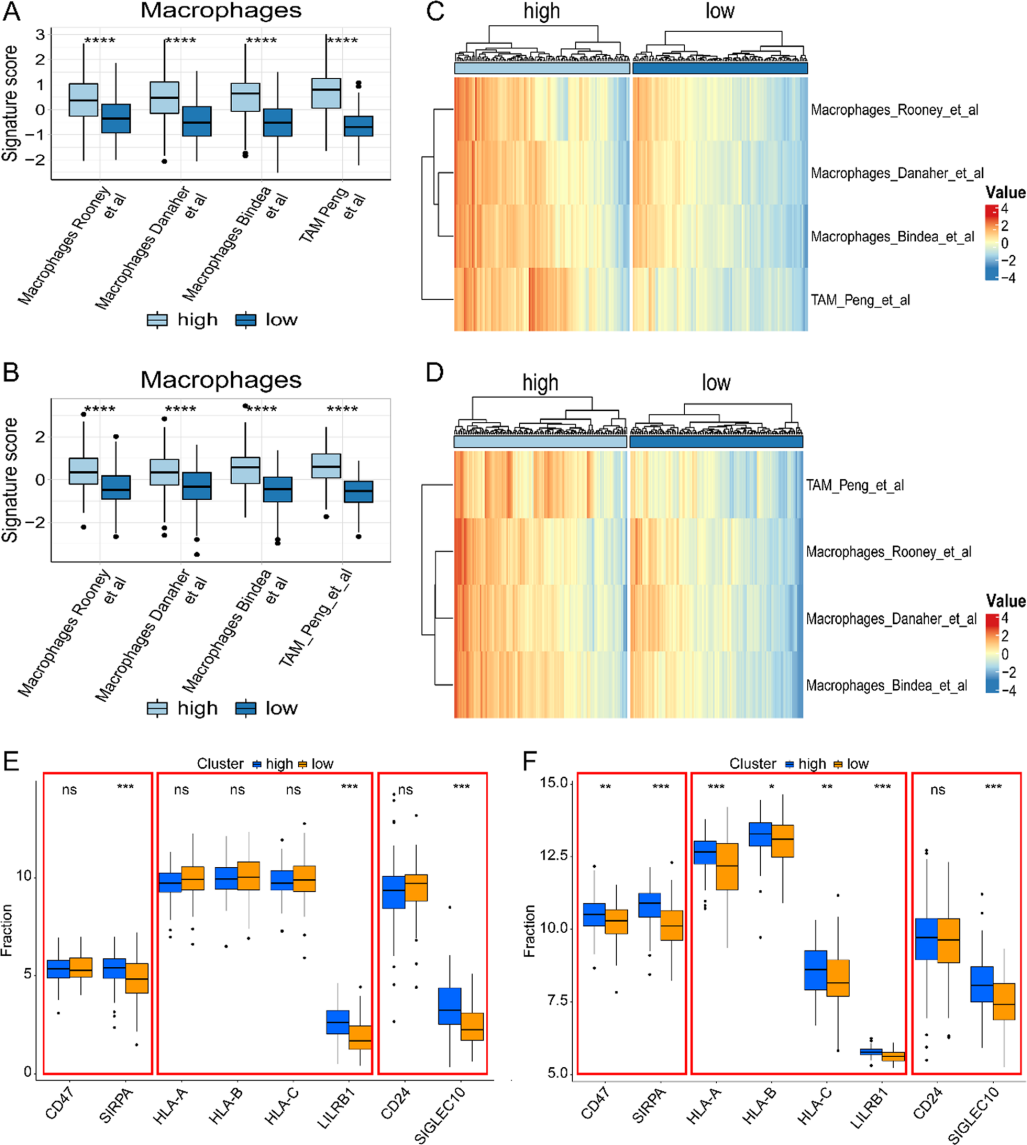
The relationship between risk-score and macrophage infiltration. The box chart shows the relationship between risk-score and macrophage infiltration in TCGA (**A**) and GSE84437 (**B**). The heat map shows the relationship between risk-score and macrophage infiltration in TCGA (**C**) and GSE84437 (**D**). The box diagram shows the relationship between risk-score and tumor associated macrophage immune checkpoints in TCGA (**E**) and GSE84437 (**F**)

In order to further explore the impact of lipid metabolism reprogramming on immune microenvironment, we calculated the tumor microenvironment score by ESIMATE algorithm. We found that HRisk group had higher immune score and stromal score, and lower tumor purity (Fig. 5A, B). Through further analysis of CIBERSORT (Fig. 5C, D and Additional file 1: Fig. S4A-B), quanTIseq (Fig. 5E, F and Additional file 1: Fig. S4C-D) and xCell (Additional file 1: Fig. S4E-F), it was found that M2 macrophages generally increased in HRisk group. In addition, the training set and validation set were further verified by macrophage scores constructed by Rooney et al., Danaher et al., Bindea et al. and Peng et al. We found that there was more macrophage infiltration in HRisk group (Fig. 6A–D). More importantly, we found that in the HRisk group, the immune checkpoints (SIRPA, LILRB1, SIGLEC10) of tumor associated macrophages involved in tumor antigen recognition disorders increased significantly (Fig. 6E, F), releasing anti phagocytic signals and negatively regulating the phagocytic function of macrophages.

### Identification of key genes based on LMAGs

**Fig. 7:**
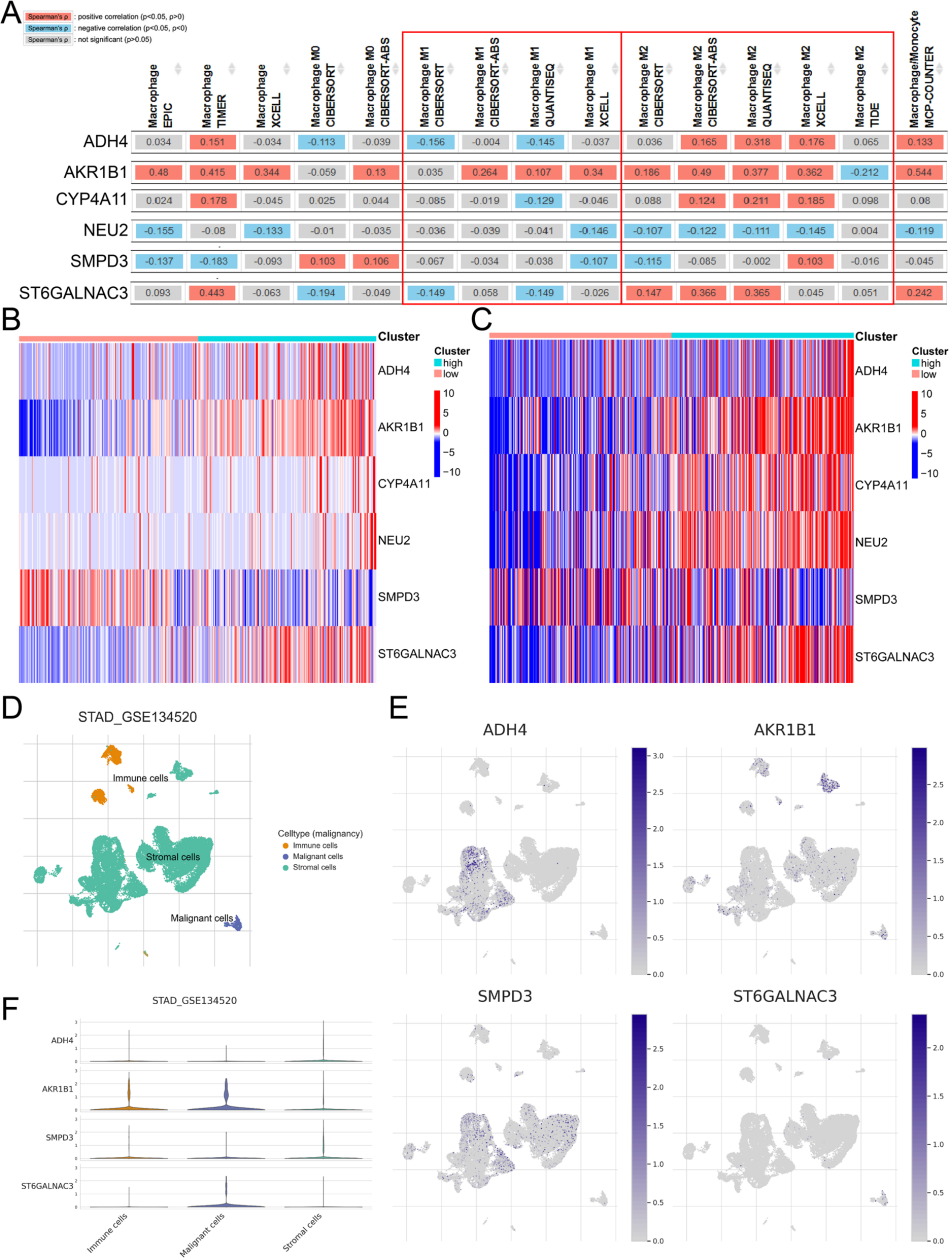
Expression of 6 LMAGs. The relationship between 6 LMAGs and macrophage is shown through TIMER2.0 (**A**). The heat map shows the relationship between risk-score and 6 LMAGs in TCGA (**B**) and GSE84437 (**C**). The expression of 4 LMAGs in tumor cells, stromal cells and immune cells was demonstrated by TISCH (**D**–**F**)

**Fig. 8:**
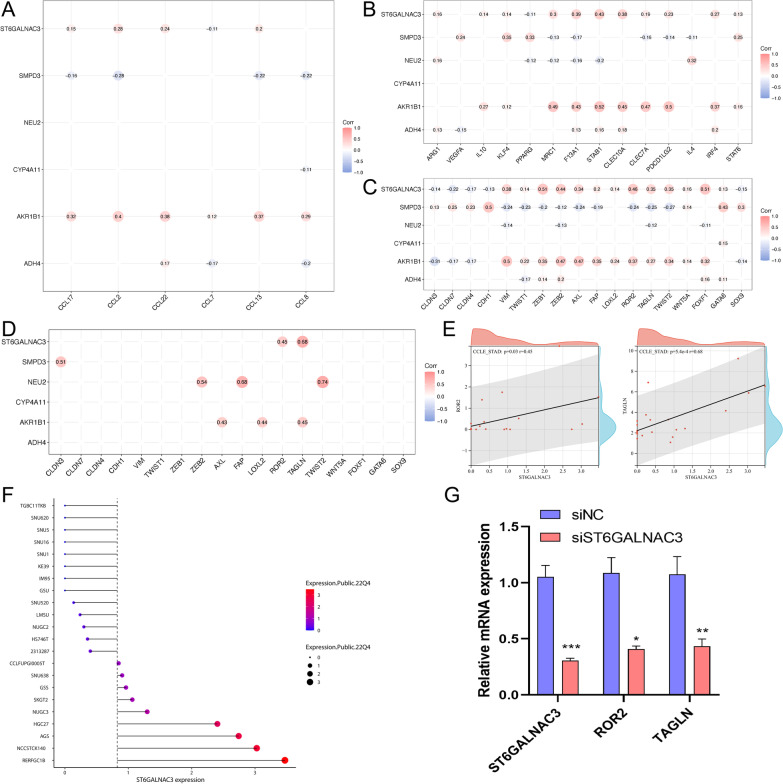
The relationship and function of ST6GALNAC3 with EMT and macrophage infiltration. The relationship between LMAGs and macrophage infiltration (**A**, **B**). The relationship between LMAGs and EMT (**C**). In the CCLE database, ST6GALNAC3 is significantly positively correlated with EMT markers (ROR2 and TAGLN) (D-E). The expression of ST6GALNAC3 in various gastric cancer cell lines (**F**). ROR2 and TAGLN decreased significantly with the knockdown of ST6GALNAC3 though PCR (**G**)

**Fig. 9:**
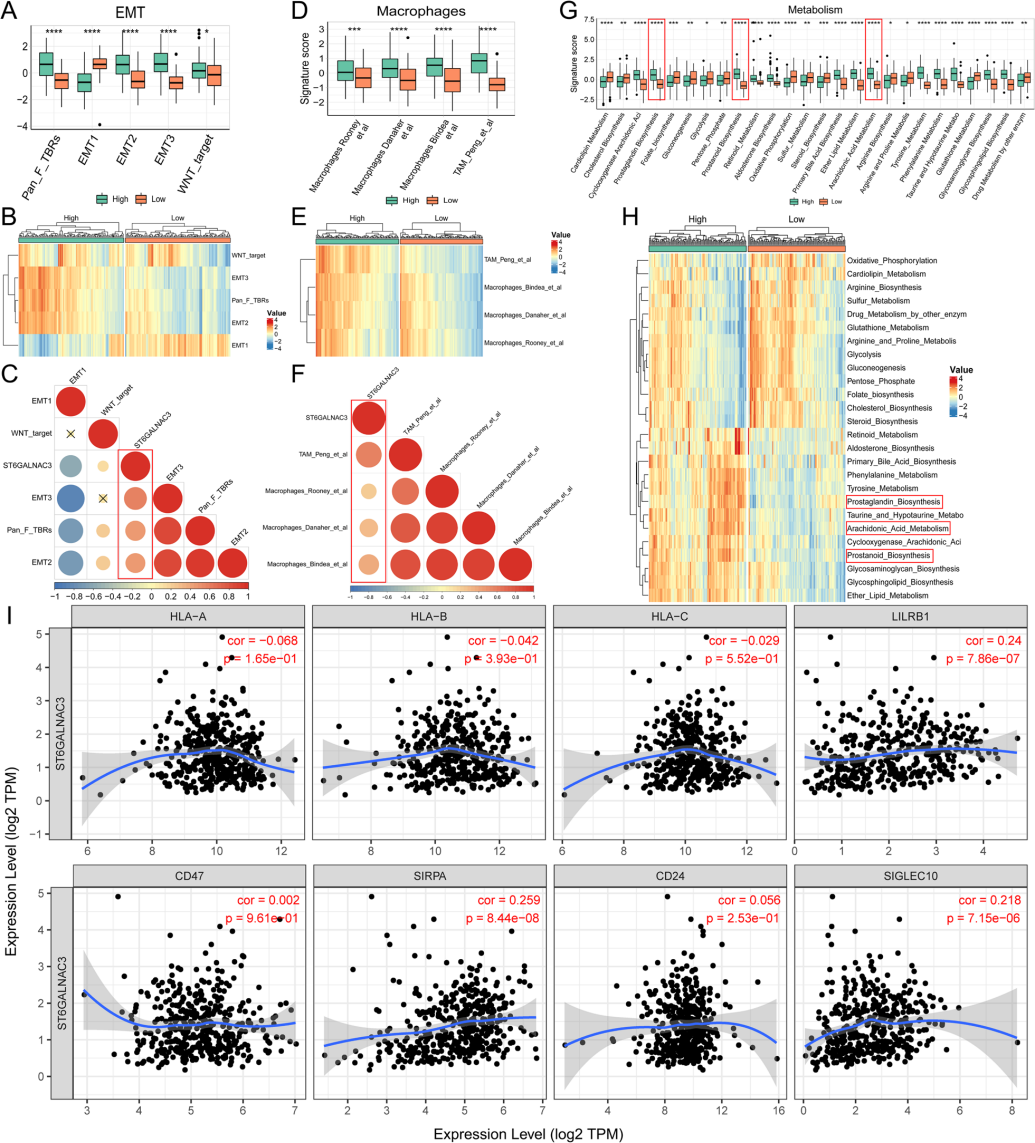
The relationship between ST6GALNAC3 and EMT, macrophage infiltration and metabolic reprogramming in TCGA. The box chart shows the relationship between ST6GALNAC3 and EMT score (**A**). The heat map shows the relationship between ST6GALNAC3 and EMT score (**B**). C shows the correlation between ST6GALNAC3 and EMT scores. The box chart shows the relationship between ST6GALNAC3 and macrophage infiltration (**D**). The heat map shows the relationship between ST6GALNAC3 and macrophage infiltration (**E**). F shows the correlation between ST6GALNAC3 and macrophage infiltration. The box chart shows the relationship between ST6GALNAC3 and metabolic reprogramming (**G**). The heat map shows the relationship between ST6GALNAC3 and metabolic reprogramming (**H**). The relationship between ST6GALNAC3 and macrophage immune checkpoint (**I**)

In order to further search for therapeutic targets related to lipid metabolism in tumor cells, we further analyzed the six genes of risk-score. Chi square analysis in the prognostic evaluation of biomarkers (Table 1 and Additional file 1: Fig. S5A-B). Through TIMER2.0 database analysis, we found that ADH4, CYP4A11 and ST6GALNAC3 were negatively correlated with M1 macrophages and positively correlated with M2 macrophages (Fig. 7A). AKR1B1 and ST6GALNAC3 were stably and highly expressed in HRisk group (Fig. 7B, C). More importantly, through single cell database (TISCH) analysis, ST6GALNAC3 is mainly expressed in tumor cells (Fig. 7D–F), and through OncoLnc and HPA database, it is found that the expression of ST6GALNAC3 significantly affects the prognosis of patients (Additional file 1: Fig. S5C-D), and the overall survival rate of patients with high expression of ST6GALNAC3 is low. ST6GALNAC3 may be a therapeutic target related to lipid metabolism in tumor cells. We also explored the relationship between LMAGs and macrophage infiltration, and found that ST6GALNAC3 and AKR1B1 significantly promoted the secretion of macrophage chemokines (Fig. 8A). LMAGs significantly promoted the expression of M2 macrophage markers (Fig. 8B). We also found that ST6GALNAC3 and AKR1B1 can significantly promote the high expression of EMT-related markers (Fig. 8C). More importantly, we verified in the CCLE database and found that ST6GALNAC3 is significantly positively correlated with EMT markers (ROR2 and TAGLN) (Fig. 8D, E). We also explored the expression of ST6GALNAC3 in various gastric cancer cell lines (Fig. 8F). Finally, we selected AGS cells for experimental verification. We found that ROR2 and TAGLN decreased significantly with the knockdown of ST6GALNAC3 (Fig. 8G). ST6GALNAC3 may promote EMT through ROR2 or TAGLN. In addition, we further investigated the effects of ST6GALNAC3 on EMT, macrophage infiltration and lipid metabolism reprogramming. We found that patients with high expression of ST6GALNAC3 had higher EMT scores (Fig. 9A–C; Additional file 1: Fig. S6A, 6D and 6G) and macrophage infiltration (Fig. 9D–F; Additional file 1: Fig. S6B, 6E and 6H), as well as higher arachidonic acid metabolism and higher prostaglandin biosynthesis (Fig. 9G, H; Additional file 1: Fig. S6C and 6F). In addition, we found that ST6GALNAC3 was positively correlated with the immune checkpoints [34] (SIRPA, LILRB1, SIGLEC10) of tumor associated macrophages involved in tumor antigen recognition disorders (Fig. 9I). In conclusion, ST6GALNAC3, as a potential target for lipid metabolism therapy, can promote arachidonic acid metabolism and upregulate prostaglandin synthesis, increase M2 macrophage infiltration, induce epithelial mesenchymal transformation, and affect the prognosis of patients.

**Table 1:** Chi square analysis in the prognostic evaluation of biomarkers

LMAGs expression	Alive	Dead	*P* value
N	227 (61%)	144 (39%)	
ADH4			0.37
High	109 (59%)	76 (41%)	
Low	118 (63%)	68 (37%)	
AKR1B1			**0.002**
High	99 (54%)	86 (46%)	
Low	128 (69%)	58 (31%)	
CYP4A11			0.97
High	113 (61%)	72 (39%)	
Low	114 (61%)	72 (39%)	
NEU2			0.1
High	80 (56%)	63 (44%)	
Low	147 (64%)	81 (36%)	
SMPD3			0.15
High	120 (65%)	65 (35%)	
Low	107 (58%)	79 (42%)	
ST6GALNAC3			**0.009**
High	101 (55%)	84 (45%)	
Low	126 (68%)	60 (32%)	

*P* values that are statistically significant are shown in bold

## Discussion

We constructed a new lipid metabolism score by univariate and multivariate analysis of lipid metabolism related genes. Then, we established nomogram by incorporating risk-score, T stage, N stage and age to predict the overall survival rate of patients. We found that nomogram has good prediction ability in both training set and verification set. More importantly, through ROC, DCA, NRI and IDI, we found that risk-score can significantly improve nomogram's predictive ability and clinical decision-making ability. The new score has good predictive ability, and nomogram based on risk-score has good clinical application prospects.

Arachidonic acid (AA) is a polyunsaturated fatty acid, which constitutes the phospholipid domain of most cell membranes and is released from cell membranes by cytoplasmic phospholipase A2 (PLA2) [35]. Free AA can be metabolized to PGE2 through cyclooxygenase (COX) pathway. In the process of tumor progression, PGE2 combines with four e-prostaglandin (EP) receptors 1–4 (EP 1–4), exerts its activity by acting on releasing cells (autocrine mechanism) and adjacent cells (paracrine mechanism), enhances tumor cell proliferation and survival, promotes angiogenesis, and induces metastasis [15, 35]. This is consistent with our study. Compared with LRisk group, patients in HRisk group have higher arachidonic acid metabolism level and prostaglandin biosynthesis level, and lower overall survival rate.

Through GO, KEGG and ssGSEA analysis, we found that HRisk group patients significantly enriched tumor invasion and metastasis related pathways and immune related pathways. More importantly, by calculating the EMT score constructed by Powles and Zhang et al., we found that the higher the lipid metabolism score, the higher the EMT score. The patients in the high lipid metabolism score have higher prostaglandin expression, and prostaglandins can induce angiogenesis and EMT, and promote tumor progression. This is consistent with recent research conclusions. It is reported that PGE2 can activate hypoxia inducible factor 1 α (HIF-1 α) [36] or cAMP signaling pathway stimulated VEGF expression [37]. In addition to the typical activation of EP receptors, PGE2 has been shown to promote cancer progression through interaction with carcinogenic signals, including epidermal growth factor (EGF) and its receptor (EGFR) [38]. In particular, PGE2 and EGF/EGFR may synergistically promote the growth, invasion, epithelial mesenchymal transition (EMT) and stem cell like phenotype of cancer cells [39]. In conclusion, our results show that risk-score can accurately distinguish patients with epithelial stromal transformation trend, help in clinical individualized treatment, and provide a theoretical basis for targeting prostaglandins to treat gastric cancer.

It is reported that patients with higher immune score and stromal score in gastric cancer have a worse prognosis [40]. This is consistent with our conclusion. We found that HRisk group had higher immune score and stromal score, and lower tumor purity score. We found that M2 macrophages in HRisk group generally increased in infiltration through a variety of immune cell infiltration algorithms. In addition, we also verified the macrophage score constructed by Rooney et al., Danaher et al., Bindea et al. and Peng et al., and found that higher lipid metabolism score would lead to increased macrophage infiltration. The analysis of immune cell infiltration showed that M2 macrophages generally increased and immunosuppressive cell infiltration increased. Although there was a high immune score, the patient had immunosuppression and had a worse prognosis. More importantly, we found that in the HRisk group, the expression of immune checkpoints (SIRPA, LILRB1, SIGLEC10) of tumor associated macrophages involved in tumor antigen recognition disorders increased significantly, and the increased binding of CD47-SIRPA [41] and MHCI/ LILRB1 [42] would lead to tyrosine phosphorylation on immune receptor tyrosine—based on inhibitory motifs (ITIMs), release the "don't eat me" signal, So as to inhibit macrophage mediated phagocytosis and protect normal cells from damage of immune system. Drugs targeting MHCI/LILRB1 axis may promote anti-tumor immune response and play a synergistic role with drugs targeting CD47-SIRPA axis; CD24-SIGLEC10 [43] interaction blocks the cytoskeleton rearrangement required by macrophage phagocytosis and triggers the inhibitory signal transduction cascade. M0 has been found to express low levels of SIGLEC10, while SIGLEC10 is strongly expressed in M2. As described in the review, the reprogramming of lipid metabolism leads to increased infiltration of tumor associated macrophages, inhibits macrophage mediated phagocytosis, and enables tumor cells to escape the monitoring and clearance of macrophages.

We further found that ST6GALNAC3 was significantly positively correlated with M2 macrophages and negatively correlated with M1 macrophages, and was significantly overexpressed in the high lipid metabolism score group. More importantly, ST6GALNAC3 is mainly expressed in tumor cells, but hardly expressed in immune cells and stromal cells. ST6GALNAC3 belongs to a family of sialyltransferases that transfer sialic acids from CMP-sialic acid to terminal positions of carbohydrate groups in glycoproteins and glycolipids. Studies have shown that ST6GALNAC3 has prognostic significance in bladder cancer [44], liver cancer [45] and lung cancer [46]. ST6GALNAC3 methylation has diagnostic biomarker potential in prostate cancer tissues and liquid biopsy tissues [47]. However, no relevant studies have reported the role of ST6GALNAC3 in arachidonic acid metabolism and immune microenvironment. Our study found that ST6GALNAC3 may be a therapeutic target related to lipid metabolism in tumor cells. We found that ST6GALNAC3 can promote arachidonic acid metabolism and prostaglandin synthesis, increase M2 macrophage infiltration, inhibit macrophage phagocytosis, induce EMT, and affect the prognosis of patients.

Our research found a novel and powerful LMAGs signature. Six-LMAGs features can effectively evaluate the prognosis of GC patients and reflect the immune status. Six-LMAGs characteristics may be involved in the regulation of immune related signaling pathways, and may provide a promising target for improving the prognosis and GC response to immunotherapy. Our results suggest that ST6GALNAC3 may be a potential prognostic marker to improve the survival rate and prognostic accuracy of GC patients, and may even be a potential biomarker of GC patients, indicating the response to immunotherapy.

## Supplementary Information

**Additional file 1: Supplementary Figure S1.** Correction of TCGA data.** Supplementary Figure S2.** The box diagram shows the relationship between risk-score and metabolic reprogramming in TCGA and GSE84437.** Supplementary Figure S3.** Verification of Nomogram.** Supplementary Figure S4.** The proportion of 22 immune cells between HRisk and LRisk was shown by CIBERSORT in TCGA and GSE84437.** Supplementary Figure S5**. Chi square analysis in the prognostic evaluation of biomarkers.** Supplementary Figure S6**. The relationship between ST6GALNAC3 and EMT, macrophage infiltration and metabolic reprogramming in GSE84437.

**Additional file 2:** The clinical characteristics of the patients.

**Additional file 3:** Univariate Cox regression analysis.

**Additional file 4:** Comparison of the NRI and IDI of the nomogram model with and without risk-score.

**Additional file 5:** Differential genes between HRisk and LRisk in GEO.

## Data Availability

The dataset was downloaded from the TCGA database (https://portal.gdc.cancer.gov/), and GSE84437 were downloaded from the GEO database (http://www.ncbi.nlm.nih.gov/geo/). All codes and data are available on GitHub (https://github.com/sanlinm/LMAG) and Figshare (https://figshare.com/authors/linm_san/14850970).
