# Correction: Construction of a lipid metabolism-related and immune-associated prognostic score for gastric cancer

**DOI:** 10.1186/s12920-023-01539-2

**Published:** 2023-05-18

**Authors:** Jing Dai, Qiqing Li, Jun Quan, Yueyi Zou, Juan Liu, Kai Gao

**Affiliations:** 1 Department of Gastrointestinal Surgery, The Third Xiangya Hospital of Central South University, 410013 Changsha, Hunan People’s Republic of China; 2 Department of Dermatology, The Second Xiangya Hospital, Central South University, 410011 Changsha, Hunan People’s Republic of China

**Correction: BMC Med Genomics 16, 93 (2023)**.

10.1186/s12920-023-01515-w.

Following publication of the original article [1], the authors would like to confirm that the author Gunther Webb is Yueyi Zou. This correction article displays Yueyi Zou’s Chinese names.

## References

[1] Dai J, Li Q, Quan J (2023). Construction of a lipid metabolism-related and immune-associated prognostic score for gastric cancer. BMC Med Genomics.

