# Supplementary material for: Construction of a lipid metabolism-related and immune-associated prognostic score for gastric cancer
Source: BMC Med Genomics. 2023 May 3;16:93. doi: 10.1186/s12920-023-01515-w (PMC10158005; doi:10.1186/s12920-023-01515-w)
Supplement: Supplementary file 1 — Additional file 1: Supplementary Figure S1. Correction of TCGA data. Supplementary Figure S2. The box diagram shows the relationship between risk-score and metabolic reprogramming in TCGA and GSE84437. Supplementary Figure S3. Verification of Nomogram. Supplementary Figure S4. The proportion of 22 immune cells between HRisk and LRisk was shown by CIBERSORT in TCGA and GSE84437. Supplementary Figure S5. Chi square analysis in the prognostic evaluation of biomarkers. Supplementary Figure S6. The relationship between ST6GALNAC3 and EMT, macrophage infiltration and metabolic reprogramming in GSE84437. [file 12920_2023_1515_MOESM1_ESM.docx]

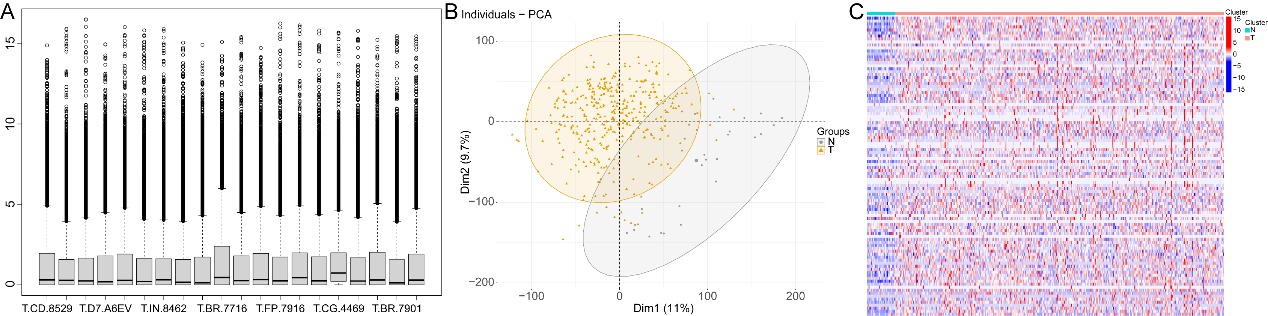


**Supplementary Figure S1. Correction of TCGA data.**


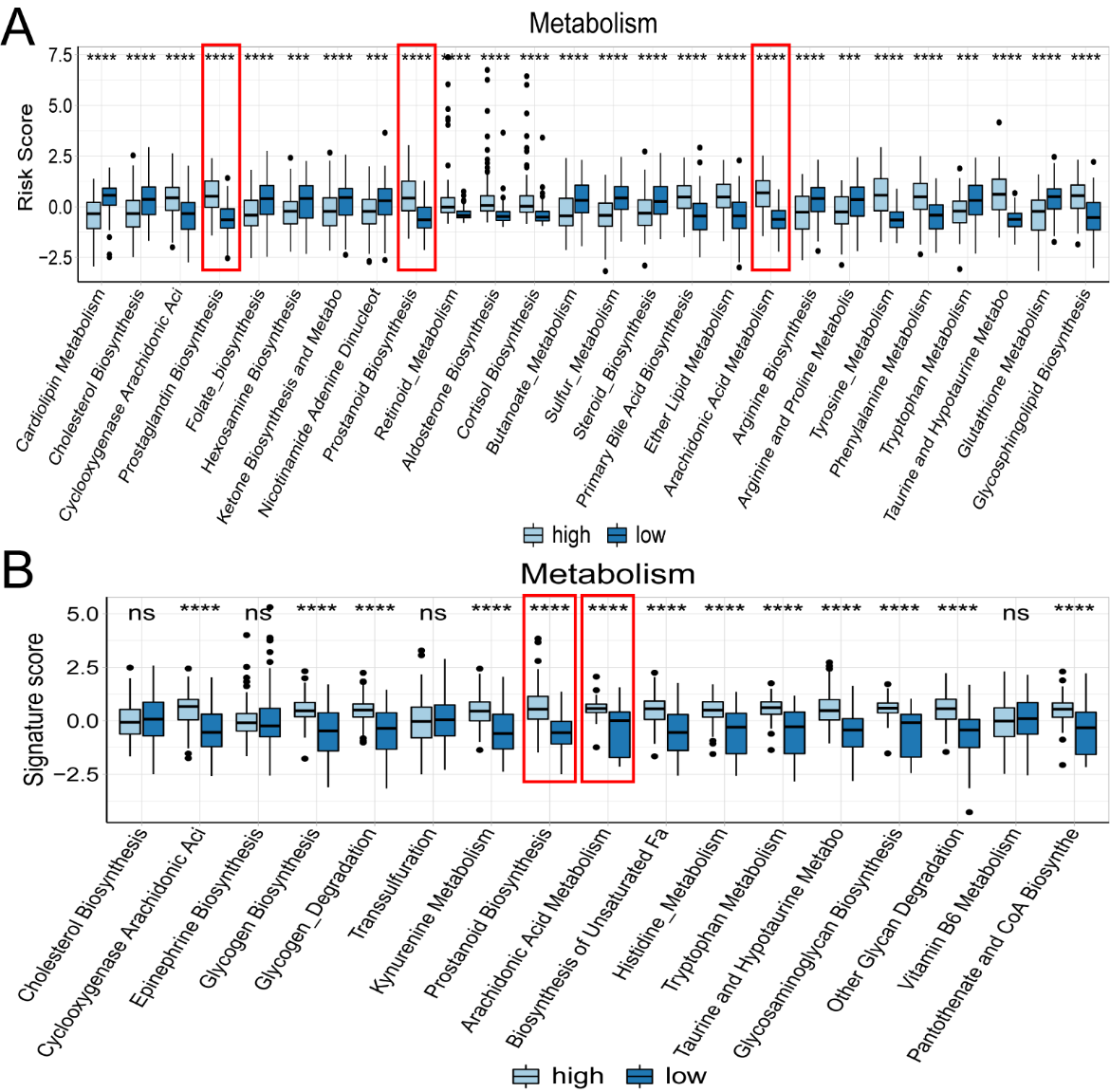


**Supplementary Figure S2.** The box diagram shows the relationship between risk-score and metabolic reprogramming in TCGA (A) and GSE84437 (B).


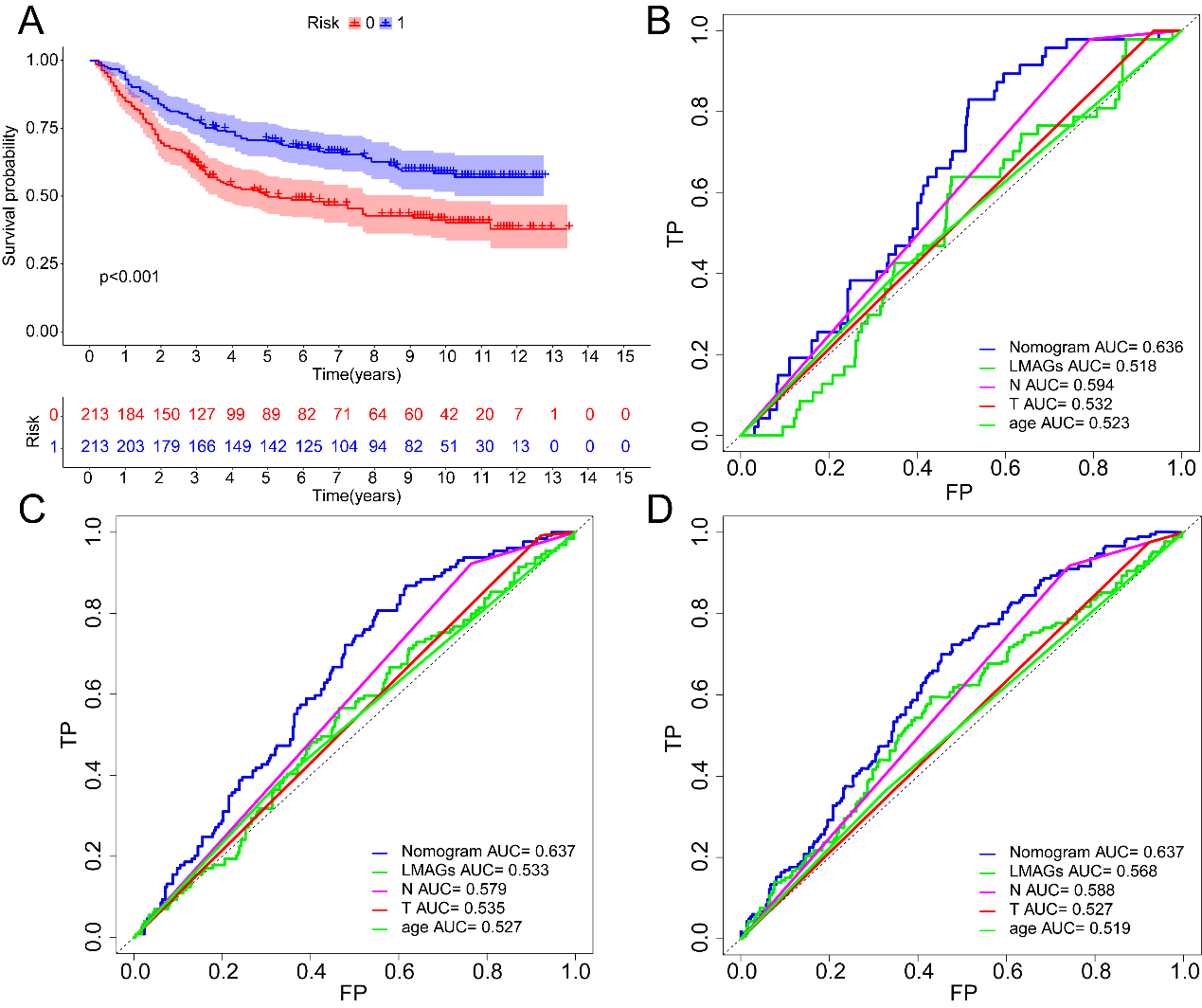


**Supplementary Figure S3. Verification of Nomogram.** Kaplan-Meier curve of Nomogram in GSE84437 (A). Nomogram ROC curve for 1, 3 and 5 years (B-D).


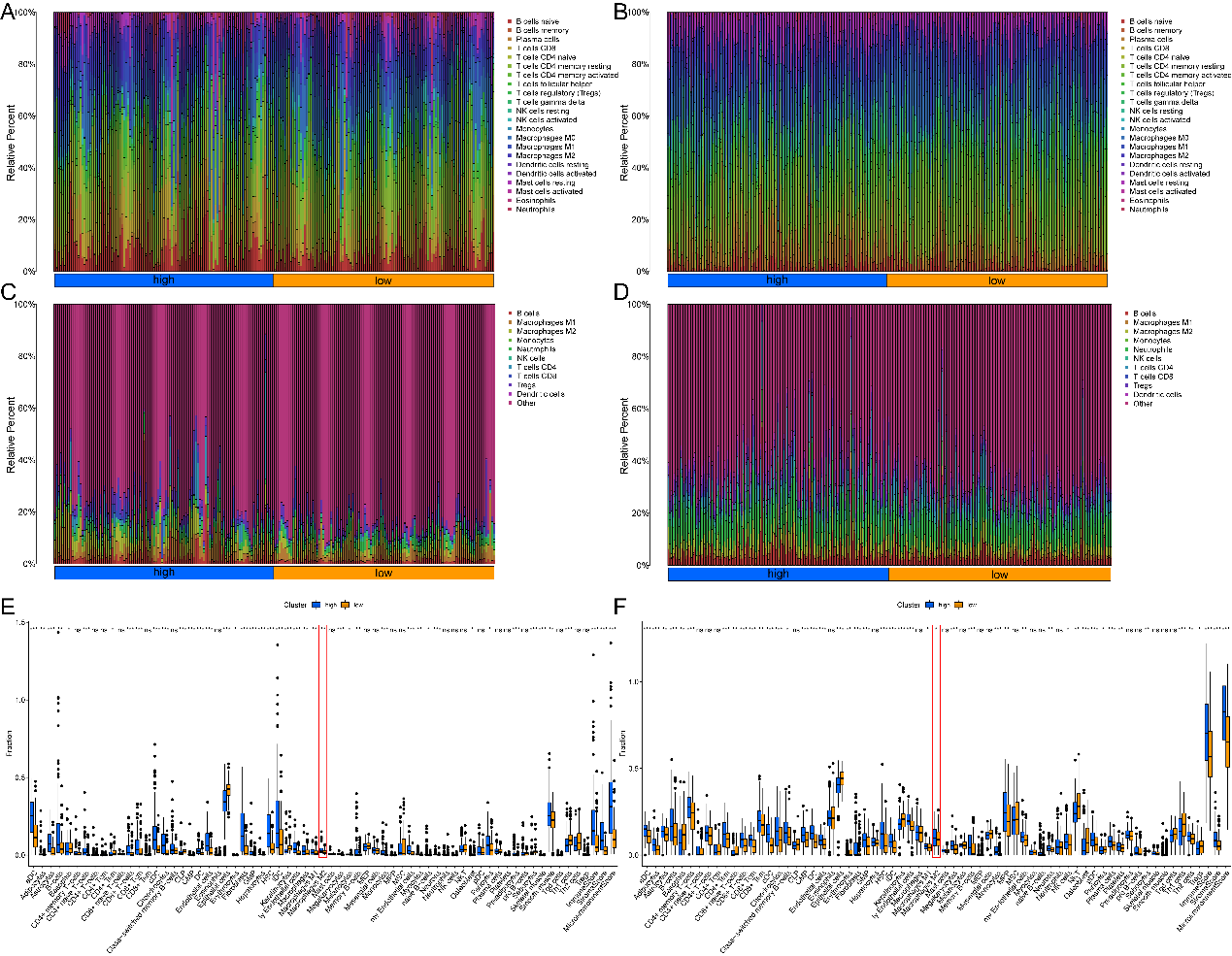


**Supplementary Figure S4.** The proportion of 22 immune cells between HRisk and LRisk was shown by CIBERSORT in TCGA (A) and GSE84437 (B). The proportion of 11 immune cells between HRisk and LRisk was shown by quanTIseq in TCGA (C) and GSE84437 (D). Boxplots depicting the xCell scores of 64 immune cells of the HRisk patients compared to LRisk patients in TCGA (E) and GSE84437 (F).


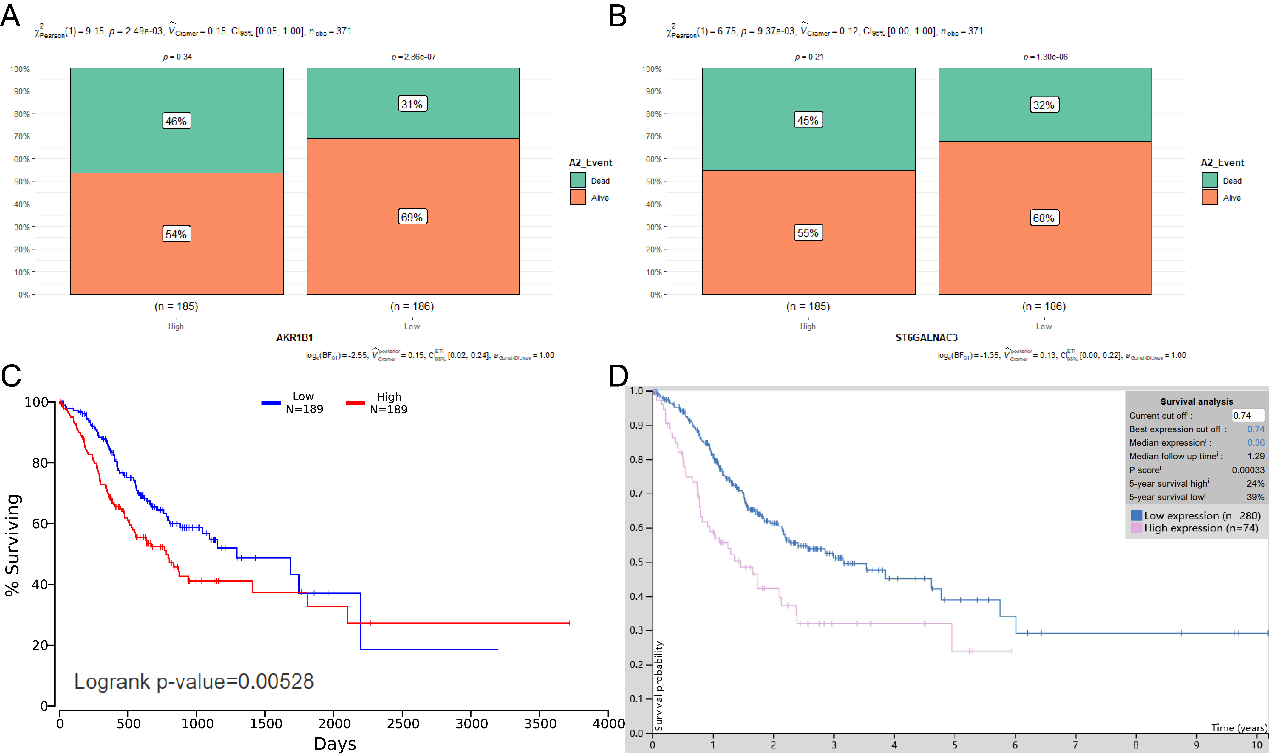


**Supplementary Figure S5.** Chi square analysis in the prognostic evaluation of biomarkers (A-B). The Kaplan-Meier curve of ST6GALNAC3 is displayed through OncoLnc and HPA data (C-D).


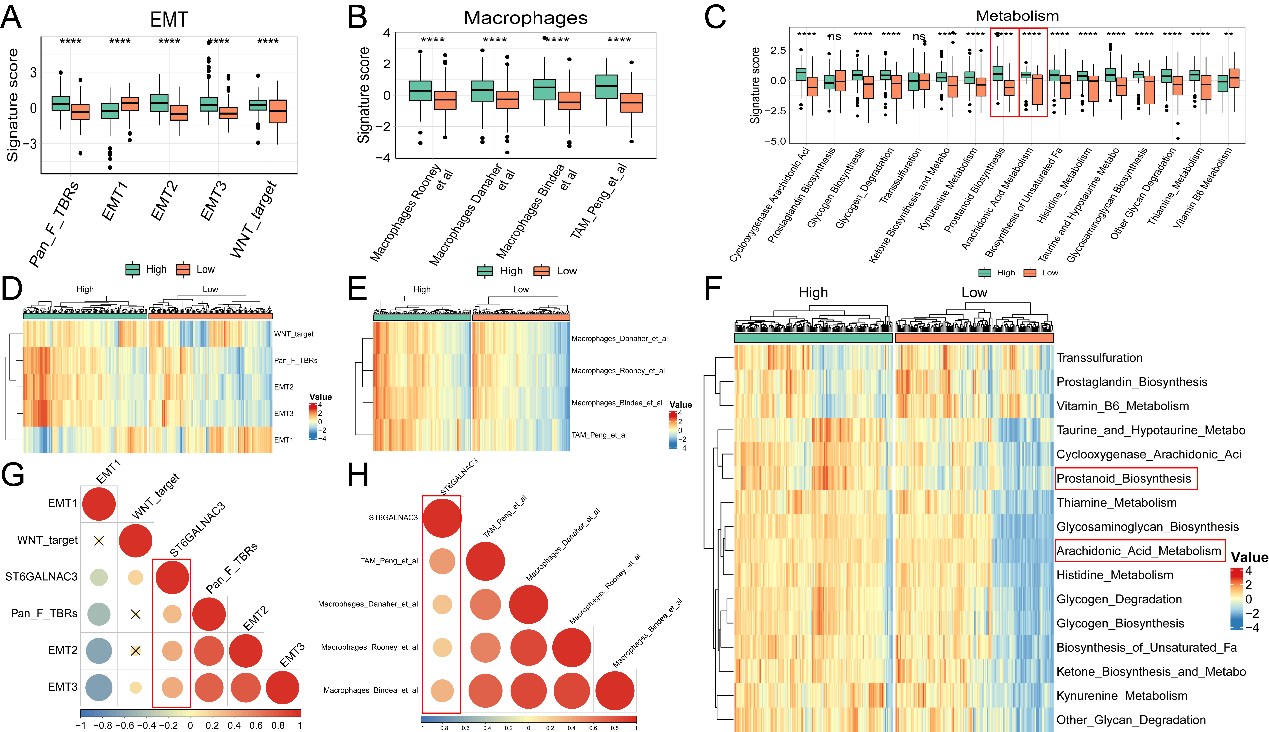


**Supplementary Figure S6. The relationship between ST6GALNAC3 and EMT, macrophage infiltration and metabolic reprogramming in GSE84437.** The box chart shows the relationship between ST6GALNAC3 and EMT score (A). The heat map shows the relationship between ST6GALNAC3 and EMT score (D). Figure G shows the correlation between ST6GALNAC3 and EMT scores. The box chart shows the relationship between ST6GALNAC3 and macrophage infiltration (B). The heat map shows the relationship between ST6GALNAC3 and macrophage infiltration (E). Figure H shows the correlation between ST6GALNAC3 and macrophage infiltration. The box chart shows the relationship between ST6GALNAC3 and metabolic reprogramming (C). The heat map shows the relationship between ST6GALNAC3 and metabolic reprogramming (F).
